# Vocal exchanges during pair formation and maintenance in the zebra finch (*Taeniopygia guttata*)

**DOI:** 10.1186/s12983-017-0197-x

**Published:** 2017-02-23

**Authors:** Pietro Bruno D’Amelio, Lisa Trost, Andries ter Maat

**Affiliations:** Department Gahr - Behavioural Neurobiology, Max Planck Institute for Ornithology, Eberhard-Gwinner-Straße, 82319 Seewiesen, Germany

**Keywords:** Monogamous songbirds, Vocal communication, Antiphonal calling, Individualized recording, Pair compatibility

## Abstract

**Background:**

Pair compatibility affects the success of a pair; however, its causes and mechanisms are not fully understood. Vocal exchange may be very important for pair formation, coordinating pair activities, maintaining the pair bond and mate guarding. To investigate the role of vocal exchange in pair formation and pair maintenance, we explored whether new and established pairs of zebra finches differed in their calling relationships. We used individualised backpack microphones to examine the entire daily vocal emission of pairs, with parallel video recording of behaviour.

**Results:**

We found that in non-breeding, isolated pairs, a specific type of call, the “stack call”, was the most common. Furthermore, all pairs used the stack call for precisely timed antiphonal exchange. We confirmed a difference between new and established pairs in social behaviour, with the former spending less time in physical contact. Notably, we found that this was mirrored by a difference in calling behaviour: members of new pairs converged over time on a more symmetric calling relationship. Additionally, we observed different response rates to partners among individuals, but a repeatable relationship of answering within pairs, which may reflect different degrees of motivation to answer the partner.

**Conclusions:**

Our findings show that there is plasticity in calling behaviour and that it changes during pair formation, resulting in a coordinated stack call exchange with a similar number of answers between partners once the pair is established. It is possible that some of the calling relationship measurements that we present reflect pair compatibility.

**Electronic supplementary material:**

The online version of this article (doi:10.1186/s12983-017-0197-x) contains supplementary material, which is available to authorized users.

## Background

Individual quality does not necessarily predict the breeding success of a pair [1–3]. Instead, pair compatibility has been proposed to influence success because of synergistic effects between pair members [4, 5]. Furthermore, in several bird species, breeding success is positively related to pair-bond duration [6–9]. Although demonstrated mainly in long-lived non-Passeriformes, the benefits of “mate familiarity” and the “costs of mate change” may partially explain the effect of pair-bond duration [10]. Pair coordination is another factor that has been shown to have fitness benefits in various songbirds [11–15], and it is possible that vocal behaviour may be important for pair coordination. However, only a few studies have explicitly examined the influence of vocal exchanges between pair members on pair coordination [16]. Despite its possible interaction with pair compatibility, vocal coordination has been mainly examined in the context of duetting. Duets represent an extreme case of vocal synchronisation, where partners adjust timing and type of vocalisations to match their mate [17]. Duets have multiple, often independent, functions in different contexts [18]: they can be directed at outsiders [19–21], and can also be important in intra-pair communication for functions such as coordination of activities and pair-bond maintenance [22, 23]. Coordinated vocal exchanges between pair members may also play a critical role during pair formation [24]. Nevertheless, the role of intra-pair vocalisations during pair formation has rarely been fully described or experimentally tested. Vocal exchange is a key factor in forming pair bonds, coordinating pair activity and maintaining pairs; it can thus provide us with an indication of pair compatibility.

Zebra finches are group-living songbirds that form lifelong, monogamous pairs in the wild [25, 26] and in captivity [27]. It has been hypothesised that they use two forms of communication, one with their partner and another with the rest of the group [28]. Zebra finches utter several thousand vocalisations each day and, with the exception of the song learned by males, the sexes have similar unlearned call types [15, 28, 29]. In zebra finches both sexes are involved in partner choice [30, 31]. Hence, behavioural coordination, potentially aided by vocal exchange using multiple call types, may be relevant for the choice of a mate and pair maintenance. The importance of song for pair formation in zebra finches has been extensively documented [32–34], and song after pair-bond establishment may be involved in stimulating the partner (i.e. females produced larger eggs with more orange yolks when paired to males with an high song output, [35]). However, song seems not to be critical for pair maintenance [36]. On the other hand, calling behaviour (e.g. the timing of calls and their interactions) and its importance in pair formation and maintenance has rarely been quantified. It is well documented that zebra finches initiate, and respond to, calls, taking turns in a vocal exchange [15, 37–39], a behaviour which is sometimes even termed duetting [40]. However, apart during environmental modification [41, 42], the importance and consistency of this alternating, antiphonal communication has not yet been assessed and high-resolution recording during pair formation is lacking.

Coordinated vocal exchanges within pairs could be achieved by assortative mating (i.e. choosing a partner because of a similar rate, or amount, of calling) or behavioural convergence (i.e. changing the calls’ temporal patterns to answer the partner). However, evidence supporting both models is lacking. Therefore, a comprehensive description of calling behaviour, both during pair formation and after a pair bond has been established, may enhance our knowledge of the mechanisms of pair formation and maintenance of bonds independently. In the zebra finch, pair formation often takes less than a week and can start within minutes [43]. To measure calling behaviour during pair formation, we chose a time period that was sufficiently long to induce a relationship, but not long enough to be confounded with nest building, reproduction, or parenting [43–45]. To precisely quantify vocal exchange, minimally-invasive long-term recordings are necessary. Individual-based recordings enable unprecedented accuracy in quantifying calling-behaviour with minimal impact on the birds [37, 46]. Here we describe the vocal processes of pair formation and maintenance considering all vocalisations of both sexes, identifying the different call types and measuring their timing.

In this study we use week-long video and audio recordings to compare established pairs with new ones. We study new dyads from the very first encounters and hereafter we refer to this group as new pairs. We examine differences in social behaviour, to determine if differences in calling patterns (e.g. the presence or the pattern of antiphonal calling) are related to pair experience. If post-pairing behavioural convergence occurs, we expect the new and established pairs to be more similar in both social and vocal behaviour at the end of the recording period. Additionally, we hypothesise that motivation to call in response to the partner, measured as the proportion of answers out of the total number of calls, may differ from pair to pair depending on pair compatibility. The motivation to answer more frequently may be in turn correlated with time spent in physical contact, linking behavioural and vocal aspects of pair commitment. With backpack microphones, we recorded individual zebra finches and their partners without interfering with their daily activities, collecting nearly half a million vocalisation events. We mainly focussed our analysis on the stack call, one of several call types in the zebra finch repertoire [15, 28], as it was the most common call produced. Stack call was initially thought to just signal movement [28], but more and more evidence suggest that it is important in an affiliative context [29] and specifically during intra-pair communication [15, 37, 39, 47]. We identified antiphonal calling using stack calls in all pairs. New and established pairs differed in the symmetry of their calling relationship in term of number of stack calls used to answer their partner; this was paralleled by differences in social behaviour. We propose that antiphonal calling with this specific call type developed during pair formation may represent a private communication channel (i.e. the meaning of the interaction is only clear to the partners), which may enhance pair maintenance and pair synchronisation.

## Methods

### Study animals and recording scheme

A total of 24 mature adult zebra finches (over 120 days post-hatch) were housed in pairs and were video and audio recorded in sound-proof chambers for one week. Inside the sound-proof chambers pairs were kept in cages of 60 × 30 × 40 cm with grit, food (egg food and mixed seeds) and water *ab libitum*. The light cycle was 13 h light and 11 h dark with the day period spanning from 8:00-21:00 hours. Birds were audio-recorded for 12 hours (8:00-12:00, 12:00-16:00 and 17:00-21:00). Maintenance (cage cleaning, replacing food and water, etc.) was done between 16:00 and 17:00 so as not to interfere with the recordings. The birds were placed in the sound-proof chamber the morning of the first day of recording. Every second day we analysed 8 hrs of recording (8:00-16:00). Established pairs (N = 4), which had all bred successfully at least once prior to the experiment, were chosen randomly from breeding facilities at the Max Planck for Ornithology during a non-breeding period to capture normal daily vocal exchange. The members of new pairs (N = 8), unrelated and randomly chosen from our facilities, had never seen or heard the partners before the start of the experiment. Just prior to the experiment, the latter group was kept for at least 7 days in same-sex groups, acoustically and visually isolated from members of the opposite sex. Half of these birds had previous breeding experience and half were naïve. After the experiment we allowed the new pairs to breed in order to verify that they could raise offspring and were thus comparable to the established pairs; all 8 newly formed pairs bred successfully. While breeding, the new pairs were equipped with dummy backpacks of equal size and weight to the recording backpack, to ensure that the equipment had not impaired copulation or any other part of the breeding phase during the experiment.

### Backpack microphone and recording selectivity

**Fig. 1:**
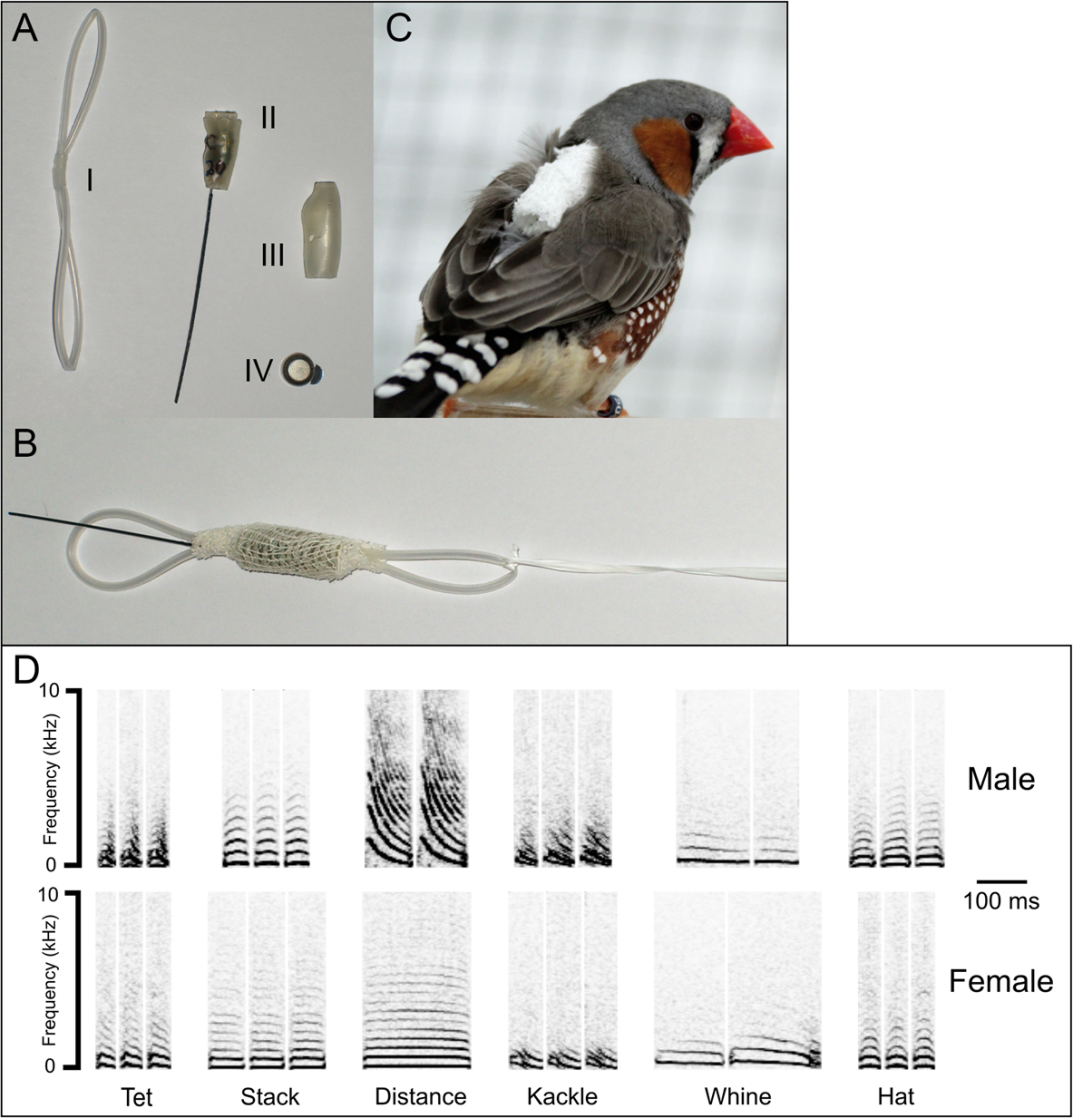
Tools: telemetry transmitter and repertoire. Top picture: **a**) Different components of the telemetry transmitter. I) Elastic cord, the upper loop encircles the head and the bottom loop goes around the tail. II) Microphone transmitter body and antenna. III) Transmitter case. IV) 1.45 V battery. **b**) The assembled backpack. **c**) Zebra finch equipped with a transmitter. Bottom panel: **d**) Sonograms of the scored calls of the male and the female from a representative experimental pair (male only: song and introductory syllables are not shown): “Tet”; “Stack”; “Distance”, “Kackle”, “Whine”, “Hat”. Despite many generations of captivity, the repertoire is very similar to the one described by Zann (1996) in wild birds. We added one soft call type, called “Hat”. The meaning and function of this call are yet to be determined, but it may be a modified version of the “Thuks” call used to indicate danger in wild populations [28]

Transmitters backpacks, and their application and employment, are described in detail elsewhere ([15, 36, 43] the specific version used in our study is the one described for males in [15]). Individuals were fitted with backpacks custom-made for each bird (Fig. 1). Briefly, transmitters were equipped with microphones (Sparrow System, Fisher III, USA), attached to a backpack and mounted on the back of each animal. The full backpacks weighed approximately 1.3 g, which is equivalent to 8.3% of the weight of an average zebra finch (15.7 g) in our colony. The harness was made of a ring of ~19 cm silicone tube (1.7 mm outer diameter, Detakta, Germany); a second 7 mm long silicone tube (1.1 mm, Detakta, Germany) was stretched and pulled over the ring, thus separating it in two loops. The audio transmitter, protected with shrinkable tubing leaving a hole for the microphone, was fixed on the narrow part between the loops with an adhesive elastic bandage (BSN medical Elastomull®haft). Finally, the transmitter was wrapped in gauze to protect it from damage and dust. One loop was placed around the neck, and one around the tail base, connected with 1.5 cm teflon tape. The posterior loop was placed rostral of the cloacal area, and the knot rested above the furcula. Backpack microphones were placed on the birds at least a week before the start of recording to allow the birds to acclimatise to the equipment [15, 46].

The AM-modulated radio signals sent by the microphones were detected using AOR8600 receivers (AOR, USA). The signal was processed in a 16-channel analogue-to-digital converter (Delta 1010, M-Audio, USA) operated at a sampling rate of 44100 Hz, and recorded using ASIO data streaming environment (Steinberg, Germany; interface adapted by Markus Kramer, MPIO Seewiesen). Each recording channel was stored as .wav file of 4 h duration.

The wireless microphone was mounted on the bird’s back, facing the body, thus primarily recording the bird’s own vocalisations [15, 37, 46]. On rare occasions, the recordings also included vocalisations emitted by other birds. However, during clustering processes these were clearly recognisable due to different basal frequency intensities, and removed [46].

### Repertoire and vocalizations clustering

**Fig. 2:**
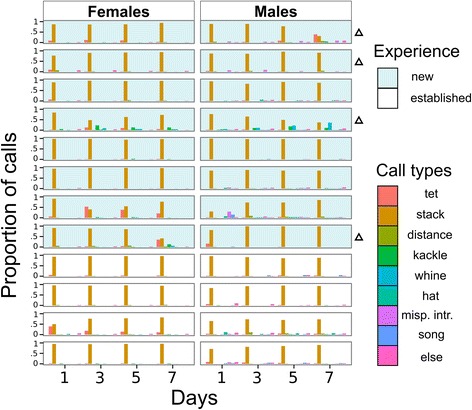
Proportion of call types by day. The proportion of each call type is reported for all the birds of the study. Each row represents a pair and the two columns are for females (*left*) and males (*right*). Within each column the 4 days of the study are plotted next to each other. The different colours of the bars represent the different call types (Misp. Intr. is the abbreviation of misplaced introductory syllables: those which were not followed by the song). The height of the bars represents the proportion of call types relative to the bird and day (sum for each bird each day equal to 1). The top 8 pairs with the shaded background are pairs that never met before the experiment whereas the bottom 4 are already established pairs. -Triangles indicate new pairs formed by individuals which had never previously bred successfully with another mate

We classified calls into different categories using previously described criteria [15, 37, 46]. Briefly, we used the custom-written software “Sound Explorer” (see [15] for GitHub address) to analyse the sonograms. For each sonogram we calculated the following parameters: duration, mean frequency, mean frequency standard deviation (SD), mode frequency, mode frequency SD, first peak, first peak SD, zero crossing, maximum positive peak and minimum negative peak. These parameters were used for automatic sorting and the output clustering was subsequently manually refined. We refined clustering using visual features of the sonograms. During the screening the scorer was aware of the treatment (pair experience). However, he/she was blind to the time information used to extrapolate data for statistical analysis. These were automatically assigned and hidden therefore not a type of subjective behavioural recording [48]. Vocalisations were classified according to the criteria described in Zann (1996), with minor modifications (Fig. 1d, bottom panel). We divided vocalisations into 7 categories: 6 types of call (Fig. 1d, bottom panel, Additional file 1) and a separate category for the vocalisations which we were unable to assign to any call type (e.g. rare vocalisations or, since they are intergrading clusters, vocalisations with features of two call types). For males we included two additional categories: song and misplaced introductory syllables (those which were not followed by the song) (Additional file 1). Zebra finches are known to include some of their calls in their song [49], therefore as first step of clustering we ordered all vocalisations in their sequence of occurrence, and were thus able to distinguish which similar call types were used in songs from those present as single calls. Finally, the number of songs was calculated dividing the total number of syllables by the average number of song syllables of each male (see Fig. 2 for birds’ daily emission of each call type).

### Video recording and scoring

The video recordings were made with small cameras (Handykam Colour 420 line CCD high resolution camera, Handykam.com, Hayle, UK) positioned inside the sound boxes but outside the cage and started automatically with a set schedule. We analysed 30 min in the morning (8:00-8:30) and 30 min in the afternoon (12:00-12:30). The videos were scored with Observer® XT (Version 10, Noldus Information Technology, Wageningen, Netherlands) with the scorer blind to the treatment. The relative position between the two birds was coded as “Clumping” if the birds were in physical contact, “Close” if the distance between individuals was less than one bird, and “Distance” if the subjects were apart (Additional file 2). Additionally, the following behaviours were scored: “Perching – exploring”, indicating that the bird was either moving or stationary in the cage; “Hopping” when the bird jumped between perches with less than 3 sec. intervals between hops, and “Preening” when the bird was cleaning its feathers (Additional file 3). Video and audio recordings were synchronised based on recognisable events (e.g. songs and/or the relative time between two vocalizations). Specifically, the audio channel of the video was extracted and aligned manually with the audio from one of the transmitters. Once synchronised and scored, the rate of each call type for each behaviour was calculated.



**Additional file 2:** Video example for each behaviour scored for the relative position. 30 seconds for each behaviour are shown. (MOV 8114 kb)




**Additional file 3:** Video example for each behaviour scored for individual behaviour. 15 seconds for each behaviour are shown. (MOV 4853 kb)


### Cross correlation analysis

We used cross-correlation analysis to determine the synchronisation of pair vocalisations [37]. The onset times of the different vocalisations were used to shape cross-correlational density plots [50], where vocalisations of one individual were aligned with specific vocalisations of individualist partner. As a convention, we designated the female calls as always beginning at time 0. The length of the time window we evaluated was 2 seconds before and after each female call, divided into 100 bins. All the calls emitted half a second before or after the call of the focal individual (the females) were considered answered and answer calls respectively [15, 37]. Answer calls are vocalisations given in response to the focal individual within 0.5 seconds. Answered calls are those that receive a response by the focal in the same time period. We used the number of calls emitted during this interval to calculate the directionality index as follows: (Number of Answers - Number of Answered) / (Number of Answers + Number of Answered). Therefore the directionality index is 0 when the number of answers is the same for males and females. The female call is the focal stimulus, thus the directionality index is positive if the number of answers is greater for the male, and negative if greater for the female. Confidence limits were calculated using Poisson probabilities based on the baseline levels of the correlation which was defined as the period between 4 and 2 seconds before and 2 and 4 after the focal vocalisations. Hence, it was assumed that calls from these two 2 seconds periods had a random distribution [37].

### Statistical analysis

Statistical analyses were conducted in R version 3.2.3 [51] using a Bayesian statistical approach with the packages “arm” [52] and “lme4” [53]. Linear Mixed Models (LMM) were calculated using the maximum-likelihood (ML) method to have a better estimation of the fixed effects [54]. Posterior means and their 95% credible intervals (CrI) were calculated (10000 simulations) using the function “sim”. We used flat prior distributions (i.e. it does not influence the posterior distribution of the simulated data), therefore sensitivity analyses of prior distributions were not required. In all cases, the residuals were checked visually for the model fit with the following plots: residual vs. fitted; residual distribution; residual variance vs. fitted. In addition, we visually checked the assumption that the random effects were normally distributed. Tables with the full model results can be found in the Additional file 4. When it was necessary to compare subgroups within an analysis we performed a derived calculation: out of the 10 000 set of simulated parameters we report the number of cases for which the estimated value of the first group was larger than that of the second group, and report this value as “p”. The threshold of 5% would be equivalent to significance level in a frequentist framework.

**Fig. 3:**
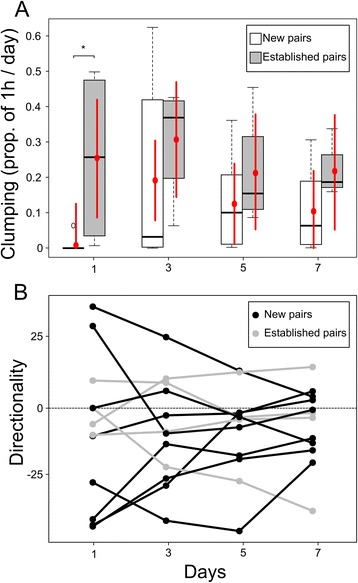
Social and calling behaviour over time. Proportion of time spent in physical contact (**a**) and directionality index (**b**) of new and established pairs over time. **a**) Amount of physical contact (“clumping”), given as a proportion of the overall time, for each of the four days. The boxplots represent the row value, the red dots the estimated Bayesian values and the red segments the Credible Intervals (CrI) estimated from the LMM. An asterisk indicates a lack of overlap between CrI and the fitted values (equivalent to frequentist significance set at <5%). **b**) Directionality index over time. Each line is a pair; established pairs are shown in grey whereas new pairs are in black. The variance in new pairs is bigger at the beginning than at the end, indicating that the relationship becomes quantitatively more symmetrical

To explain the amount of time spent in physical contact, termed clumping, we included the experience of the pair (categorical, 2 levels) and the day (categorical, 4 levels) as explanatory variables in the LMM, and since we expected that the effect of familiarity changes with time (days), we included the interaction. Pair ID was added as random factor (categorical, 12 levels) (Fig. 3a). Clumping ~ Experience * Day + (1|PairID)

To study the directionality index over time of new and established pairs we ran a LMM with experience and day as explanatory variables. We used the absolute value of the index because we focused in its difference from 0 and not its direction. Because we were interested to know whether the two treatments changed over time we included the interaction between pair experience and day (Fig. 3b). |Directionality| ~ Experience * Day + (1|PairID)

To determine the relative distance at which birds vocalised most frequently, we used a LMM with relative position as the explanatory factor (categorical, 3 levels) of the calling rate. For this model we only considered the calling rate of stack calls. The square root of the calling rate was taken, to achieve normal distribution of the residuals. Pair ID and day were used as random factors to account for repeated measures (Additional file 5). Calling rate ~ Relative position + (1|Day) + (1|PairID)

**Fig. 4:**
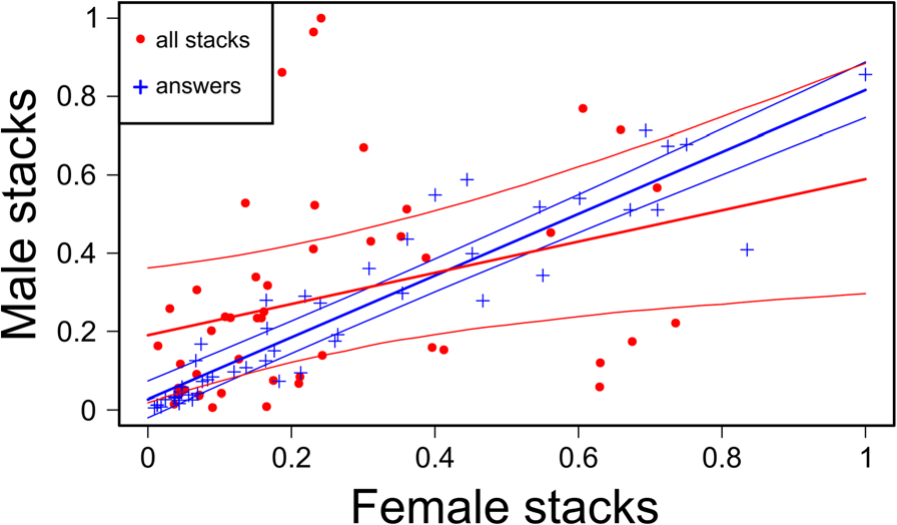
Correlation of the numbers of stack calls and answers between males and females. Total number of stack calls (*red dots*) and number of stack call replies to stacks (*blue crosses*) for males and females for each day for each pair (values are normalised dividing by the highest number of calls). Bold lines represent the relationships extracted from the estimated posterior of the linear mixed models; thinner lines are the respective 95% credible intervals. Number of answers is tightly correlated between males and females, while call number is only loosely correlated, indicating that different individuals use differing proportions of calls to answer the partner

To study the correlation of the numbers of stack calls between males and females we used 2 LMMs considering either the males’ total number of calls or the males’ number of answers as outcome variables and the corresponding females’ variables as explanatories. Both models had day of recording and pair experience nested into pair ID as random factors (Fig. 4). To represent the two models in the same plot we normalized the data dividing, for each relationship, by the highest number of calls. Total male calls ~ Total female calls + (1|Day) + (1|Experience/PairID); Male answer calls ~ Female answer calls + (1|Day) + (1|Experience/PairID)

**Fig. 5:**
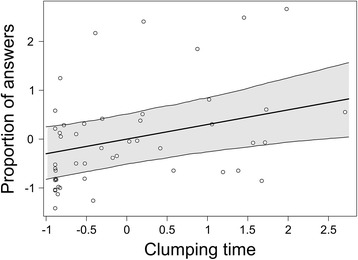
Correlation between proportion of male answers and time spent in physical contact. Amount of time in physical contact and proportion of answers (standardised by z-scores). The dots are the raw values, one point for each male each day, the bold line is the fitted regression line drawn from the posterior distribution of the value estimated from the LMM. The thinner lines and the shaded area represent the credible intervals

To determine if vocalisations were related to behavioural aspects, we modelled the proportion of calls used as answers (out of the total number of calls of the focal individual) as a function of the time spent in physical contact (Fig. 5). We ran 2 separate models for the 2 sexes. For these LMMs, we used the same random effect structure as the models of stack calls described above. Additionally, since measurement units were different and measurement values were several orders of magnitude apart, we standardized both variables using z-scores to simplify the interpretation. We also ran the same model excluding the null clumping values, days in which birds did not clump, to confirm the result. Percentage of answers ~ Clumping + (1|Day) + (1| Experience/PairID). The repeatability of the directionality index was calculated according to Lessells & Boag (1993) [55], where the among-groups variance component describes variance among pairs and the within-group variance component describes the variance within a pair across different days.

## Results

### Proportion of different calls by day

We first looked at the proportion of different call types emitted by individual birds exposed to our experimental conditions. We recorded and categorised 475 903 vocalisations. Only a small portion of the vocalisations (mean ± SD per recording; 2.62 ± 2.43%, N = 96) were not assigned to one of the depicted call types (Fig. 1d). In 94 cases out of 96 (12 pairs recorded for four days in a week) the stack call was the most frequently emitted call type (0.84 ± 0.16%, N = 96) (Fig. 2, Additional file 6). This was the same in both new (8-hour recordings where stack calls were the most common call type / total number of recordings; 62/64) and established (32/32) pairs, and it did not change during the observed period (day1: 24/24; day3: 23/24; day5: 24/24; day7: 23/24). Stack calls were almost always the most frequently-emitted call in both new and established pairs.

### Social behaviour of new and established pairs

We asked whether the proportion of time spent in physical contact (clumping) differed depending on pair experience. We found that new pairs spent very limited time clumping during the first day (time of clumping expressed in seconds and as percentage of the total time scored, mean ± SD; 29 ± 81 sec., 0.8 ± 2.2%, N = 8) (Fig. 3a), whereas established pairs clumped for much longer (919 ± 923 sec, 25.5 ± 25.7%, N = 4). Credible intervals do not overlap zero, indicating a marked difference (899 sec., CrI: 181 – 1620, p = 0.0032). In the following days, the trend that new pairs spent less time clumping continued, but the credible intervals of new and established pairs overlapped (Fig. 3a) indicating that there was no longer a clear difference (day3: p = 0.0832; day5: p = 0.1409; day7: p = 0.0817). Only the established pairs spent a considerable amount of time in physical contact during the first day, whereas the newly introduced pairs spent less time in contact.

### Calling behaviour of new and established pairs

To see if new and established pairs differed in vocal coordination, we used cross-correlations to show whether there was a specific answer to our focal stimulus, the partner calls. Pair members used different combinations of call types to respond to their partner, and these combinations of call types showed a coordinated pattern of replies (i.e. over-threshold number of calls within the analysed time window, see method “cross-correlation”) (Additional file 7). However, only one combination, stack-stack, was present among all the studied pairs on each day (Additional file 7, Additional file 8). We confirmed that mates answer to each other with very precise latency and low rate of overlapping calls [37] (Additional file 8). Observing the stack-stack calling we found that both new and established pairs tended to respond to their partners, with pairs varying in the number of replies (antiphonal calls) and total calls (Additional file 6, Additional file 8). The shape of the cross-correlation histogram, which shows the amount of replies of the partners compared to baseline calling, can therefore be used to describe the calling relationship (Additional file 8). The shape can be summarised by the directionality index, which changed from pair to pair (some pairs were asymmetrical, others were symmetrical), and also over time within the pairs. We found very high repeatability (r ± SE: 0.94 ± 0.03, *N* = 12) in the directionality index, indicating that each pair develops a specific calling relationship. The directionality index values (Fig. 3b) of new pairs were very wide in range on the first day (mean ± SD, day 1: -12.27 ± 32.64, *N* = 8) and tended to converge to a more symmetrical relationship over time (day 7: -5.90 ± 10.21, *N* = 8). The absolute value of the directionality index statistically differed between the first and the last day (*p* < 0.0001, *N* = 8). In contrast, the index of established pairs did not change significantly (day1: -1.43 ± 8.74, day 7: -7.05 ± 22.32; *p* = 0.1011, *N* = 4). Furthermore we observed a more symmetrical relationship of established pairs compared with new ones during the first day (probability that new pairs had higher directionality index than established ones, *p* = 0.0006, *N* = 12); the difference was not significant during the last day (*p* = 0.8084, *N* = 12).

### Correlations of total number of stack calls and reply stack calls

We defined motivation to answer the proportion of calls used as answers out of the total number of call emitted. Consequently, to understand whether the motivation to reply differed within and between pairs, we compared the total number of stacks between partners with the proportion of the number of stacks used as replies (to other stacks) (Fig. 4). If the two distributions were similar it would mean that each individual used the same proportion of calls to answer the partner (i.e. the motivation to answer was similar among individuals). In contrast, we found that the two relationships differed greatly in shape and dispersion, indicating that each bird answers to the partner with a different proportion of calls. If the slope of the relationships were 1 and the intercept 0, it would mean that the number of calls, either the total calls or only the replies, was equal between males and females. On the contrary we found a difference between males and females in the total number of calls (estimated regression line; y = 0.190 + 0.398x). However, when considering the number of replies, the number of calls used was more similar (y = 0.026 + 0.790x). Most interestingly, if the credible intervals (CrI) were narrow it would indicate the use of a similar strategy across pairs. We found that this was the case for the number of answers (0.711 - 0.870), which was very similar between and across pairs, whereas the total number of calls had a wide CrI (0.066 - 0.739) and was only loosely correlated between and across pairs. To further explore the difference between the correlation of number of answers and total number of calls we measured the goodness-of-fit of the models, marginal and conditional r^2^-values (i.e. how much of the variance is explained by fixed effects alone and total respectively; [56, 57]). We found that for total amount of calls, marginal (r^2^m) and conditional (r^2^c) r^2^-values were 0.102 and 0.777, whereas for the number of replies, r^2^m = 0.860 and r^2^c = 0.943. We found that the experience of the pair did not explain any variance and most of the variance explained by the random factors was due to differences between pairs. Furthermore, for the model including the total number of calls, the residuals against the random factor “day” showed a specific pattern. This probably was because they called much less during the first day than predicted from the model.

### Relationship between clumping time and proportion of answers

As we found that different individuals answer with different percentages of calls (Fig. 4) we tested the correlation of vocal with the social behaviour. We combined information from the video and audio recordings to calculate the relationship between the time spent in physical contact (mean ± SD expressed in seconds; 556 ± 627 sec., N = 12) and the proportion of replies of the males’ stack calls (expressed in %, 15 ± 10.1%, N = 12) (Fig. 5) and of the females’ stack calls (17.7 ± 10.8%, N = 12) on their total number of stacks. A higher proportion of calls used as answers might reflect a higher motivation in answering, and also a longer time spent in clumping might reflect a stronger motivation to stay in contact. We found a positive relationship between standardised time spent clumping and the proportion of replies (after standardisation, see “statistical analysis” in methods, slope: 0.300, CrI: 0.097 - 0.499; Fig. 5). This means that each increasing unit of clumping time (expressed as change in standard deviation) yields an increased expected proportion of replies by about a third of a unit. For instance, an increase of 627 sec. of clumping time would lead to an increase of 3% in the proportion of calls that a male uses as replies. The number of 0 s in the clumping values may bias the model. Interestingly, also excluding occurrences in which the birds did not clump, yielded to a very similar result (slope: 0.282, CrI: 0.073 - 0.492). For the proportion of calls used by females as answers, this relationship was not as strong (slope: 0.068, CrI: -0.182 - 0.315). Interestingly, the experience of the pair did not explain any variance in the proportion of answers of male or female stack call. We conclude that clumping time can predict, to a certain extent, the proportion of replies of the male.

## Discussion

Antiphonal calling with stacks was a common feature for all zebra finch pairs in this study; this alternating calling behaviour was temporally precise, characterised by a very low rate of overlap between calls and a high level of alternation between mates. As both new and established pairs displayed this behaviour, we propose that this pattern of alternating stack calls could define a private channel of communication between mates in non-breeding situations, possibly a display of monogamous pairs [58]. Physical contact, termed clumping, has been used by many studies as a behavioural indicator of bonded pairs [59–61]. As expected, when comparing new and established pairs, we confirmed that only the latter spend time in physical contact during the first day [43]. Interestingly, the difference in social behaviour between new and established pairs is mirrored by a difference in calling patterns. We found that both new and established pairs exchanged stack calls; however, new pairs were more variable with regard to the directionality of the calling relationship during the first day (i.e. often the relationships are asymmetrical, meaning that one member answered more than the other). A week later, these new pairs had more symmetrical calling relationships. We can describe the observed pattern as behavioural convergence, labelling it as post-pairing adjustment [62]. The number of stack calls of males and females within a pair was loosely correlated, whereas the number of calls used to answer the partner was similar between pair members. This suggests that quantitatively pairs had a balanced vocal exchange, but each bird used a specific and different percentage of calls to answer the partner. The percentage of replies (‘answer calls’) by the males positively correlates with the amount of clumping exhibited by the pair. We tentatively interpret this as stronger motivation towards the partner expressed by both vocalisation and affiliative behaviour. The vocal exchange of stack calls did not occur when the birds were clumping, but rather when they were distant from each other. This suggests a function of vocal exchange during locomotion using this call type, perhaps when birds are relatively close [28, 29].

Previous studies have already described antiphonal calling involving stacks [15, 37]. Here, we added a detailed and quantitative description of the pattern of stack call usage during establishment of new pairs and the consistency of stack usage over time in already established pairs. High behavioural similarity between partners may make cooperation more effective, and may have fitness consequences in species with bi-parental care (part of the “mate familiarity effect”, reviewed [10]). Coordination of other behaviours, such as provisioning and foraging, has been found to be beneficial in zebra finches in the wild [13]. The antiphonal exchange might aid the coordination between partners, and possibly improve decision making processes (e.g. during foraging behaviour). Alternatively, or additionally, as a display that continues after the formation of the pair bond, it could be important for pair maintenance [58, 63], or potentially support mate guarding. However, whether the symmetrical communication has a functional value is still an open question. Experiments measuring fitness parameters are necessary to answer it.

We observed a large difference between marginal and conditional r^2^-values in the model correlating the total number of calls of males and females. The pattern of the residuals vs. random effect “day” did not follow a normal distribution and partially explains this result: the daily number of calls changed during the experiment and changed differentially for males and females. On the contrary, the random factor “day” did not explain any variance in the model correlating the number of answers. Therefore, showing that the answering relationship was acquired early in the pair development and the proportion of answers remained consistent over time despite the change in the overall amount of calls. The pair forms quickly within the first days [45], and we showed that in conjunction, the vocal relationship stabilized early. This partially explains why the experience of the pair did not influence either the relationship between number of calls and number of answers or the relationship between the latter parameter and the amount of clumping. However, the very high variability between pairs and the small sample size might mask the differences between groups. From the analysis comparing number of answers and number of calls it is possible to draw further conclusions. We observed a high behavioural similarity between paired males and females in the number of calls used to answer the partner [37]. However, when we considered the total amount of calls produced, we observed asymmetry between partners. This might reflect a different motivational state and interest of the birds towards their partner [64], since in our experimental design, individuals could not choose their partners. The quality of the match might therefore differ substantially among pairs, producing different patterns of calling and replying. In addition, we found that clumping time predicts the percentage of answers of the male. Males that spent more time in physical contact also used a higher proportion of their calls to answer their partner. Hence antiphonal calling could be tested as an indicator of pair compatibility. To find methods to quantify pair quality, compatibility, is very important since fitness can depend on it [2, 5]. Further, specifically designed experiments involving mate choice are needed to better clarify the relationship between answering rate and clumping. Likewise latency to the first occurrence of affiliative behaviours, such as clumping, allopreening, copulation, and their quantity, can be studied in correlation to fitness and vocal behaviour to find what factors better predict pair compatibility.

The vocal repertoire was similar among different birds and the most common call type for isolated pairs in a non-breeding situation was almost invariably the stack call. Different authors have reported other calls to be the most common; Zann (1996, ch. 10, p. 197) described the tet, others the distance call [65, 66]. This could be due to the context in which the recordings were made, or the tools used to record vocalisations. For example, the cited studies used an external microphone that might have failed to detect vocalisation with low amplitude [65, 66]. Also, the birds used by Zann were in groups and allowed to breed. That breeding status affects the type of calls that are emitted is supported by Gill et al. (2015), which shows a change in abundance according to the context; this could alter the relevance of some calls according to the deviance (i.e. the relative abundance of a particular call type [67]). Furthermore, Gill et al. (2015) showed that in a group situation, in contrast with our isolated pairs, the stack call is not always the most common type and other call type combinations other than stack-stack were always present between pair members. These differences suggest a social meaning for other call types and call combinations (i.e. some calls and call combinations might have a message for members of the group other than the mate).

Given the diversity of calls and their different uses, studying the temporal relationships of vocalisations could improve our understanding of complex communication [68] and the ‘linguistics’ of calling. Most language usage is interactive, involving rapid turn-taking characterised by short turns and very rapid responses [69]; zebra finch vocal exchange mirrors this pattern. The zebra finch calling system is clearly lacking flexibility in its messages compared to that of humans; however, turn-taking patterns and tempos of the different systems can be compared. Hence investigating the dynamic pattern of calling could help to understand the role of turn-taking in vocal communication [70]. The first step in this direction would be to verify that these calls are enough to identify the caller, making possible to select the interlocutor. Moreover, this fast exchange model of vocal communication can be investigated from the point of view of behavioural neurobiology [37]. During antiphonal calling, a bird must provide the specific appropriate response within a few milliseconds of an auditory stimulus. When the bird hears a call, it needs to process it: that is extract the type of call and the calling individual’s identity, recall the memory of that individual, and choose and utter an extremely rapid response, which makes our system ideal for investigating processing recognition and answer choice.

Our approach, with the use of backpack microphones and continuous recording, allowed an extremely high level of precision and accuracy in our measurements. However, despite the high repeatability of the turn-taking behaviour, due mainly to the time consuming procedures, the sample size is a limitation of our study, and it is therefore difficult to confidently generalise all of our results. Nevertheless, we are convinced that the results and the approach presented here may spur further research on calling patterns because of its relevance for different fields.

## Conclusion

Here, we document differences between new and established zebra finch pairs, shedding light on the role of alternating (antiphonal) calling. Members of both new and established pairs use stack calls to answer their partner. While birds in established pairs respond to their partner with equal number of calls, the newly formed pairs begin with one bird calling more, but then develop a calling relationship that becomes more symmetrical over time. We therefore found post-pairing behavioural convergence between pair members, whereby they adjusted the number of calls used to answer their partner. In addition, within both groups, pairs differed from each other, but were internally consistent. Furthermore, in males, reply frequency was positively correlated with the time spent in physical contact with their partner. The high repeatability together with the possible reflection of a motivational state leads us to postulate that the study of such calling relationships might add information on pair compatibility. We propose that the patterned exchange of vocalisations may represent a fundamental part of the pair bond, and may serve as a private channel of communication within the pair.

## Additional files

Additional file 1: Audio examples of the call repertoire of the zebra finch. (same pair as Fig. 1). Five calls for each sex and call type are spaced by one second silences. We randomly selected calls to be presented from the ones not containing noise. Sounds were recorded with backpack microphones and their amplitude normalised to -0.1 dB (maximal sample value). (7Z 240 kb)
Additional file 4: Tables with estimates, Standard Errors (SE), Credible Intervals (CrI) and random factor and residual variance of each LMM used. First is presented model structure, together with mean and SD of the raw data. The graphical representation, if present, is referred after the model. (DOCX 24 kb)
Additional file 5: Figure S1. Rate of stack calls during different relative position of pair members. Rate of stack calls (n/sec) for the 3 different relative positions scored. Clumping: the pair is in physical contact. Close: the space between the birds is less than one bird. Distance: the birds are apart. Boxplots are drawn using raw data, the red dots are the estimated Bayesian values and the red segments the Credible Intervals (CrI) estimated from the LMM.. Both males and females used different rates of stack calls depending on their relative position (data not shown for each sex separately). While in physical contact, (clumping), the birds called the least (measured in calls/sec., mean ± SD, 0.090 ± 0.115 calls/sec., N = 12), followed by close proximity, (close), (0.160 ± 0.126 calls/sec, N = 12); whereas when they were spatially separated, (distance), the pairs had a higher rate of calling (0.243 ± 0.183 calls/sec., N = 12). Using the output of the LMM we calculated the probability that estimated values of one of the relative positions would be higher than the ones of another; asterisks indicate p < 0.05. We found that the probability that the calling rate during “Clumping” was higher than “Close” was p = 0.0074, and “Close” higher than “Distance” was p = 0.0556, indicating strong differences between these categories. Thus, relative position influences the amount of elicited calls. (PNG 333 kb)
Additional file 6: Table S1. Total and proportion of different call type by bird. Total number and proportion (in brackets) of each call type for each individual. The total number of calls is the mean of the four days of recording and the proportion is calculated from this mean. Misp. Intr. is the abbreviation of misplaced introductory syllables (those which were not followed by the song). (XLSX 11 kb)
Additional file 7: Table S2. Response strength for each pair each day each combination. Within a time window of interest of 4 s before and 4 s after call onset for our cross-correlation histograms we counted the number of calls. We divided the time window with a binwidth of 50 ms (i.e. 160 bins in total). The number of calls in the bins in the first 0.5-s (Nbase, i.e. the calls between 4 and 3.5 seconds before the focal calls) was used as baseline and those in the 0.5-s bins after call onset (Nresponse) as the response. We calculated the response strength index for each call combination for each pair for each day as follows: Rresponse = (Nresponse –Nbase) / (Nresponse + Nbase). The index range between -1 and +1, positive values correspond to an increase of calling after the stimuli (partner calls) compared to the baseline, negative value to an inhibition of calling, values close to 0 to maintenance of baseline calling (all the values are multiplied by 100 to aid readability). We set a threshold to avoid weak correlations to bias the results: if the number of calls within the time window considered did not reach 160 (i.e. one call for each bin) the index was not computed (reported as 0). (XLSX 31 kb)
Additional file 8: Figure S2. Stack-stack cross-correlation for each pair. Each row represents a pair, and each column shows a different day of the experiment. For each scored day per pair, a cross-correlation graph [50] is presented of the stack-stack call relationship between male and female. Cross-correlation histograms show the temporal correlation between one male and one female call type within a given time window. Histograms were aligned on female vocalisations. The y-axis represents the number of calls, normalised by the bin with the highest number of occurrences (between 0 and 1). The interval considered on the x-axis is 0 ± 2 sec. The 0.99 Poisson confidence limits are shown with horizontal red lines [37]. Typically, the shape of the histogram is characterized by a sharp inhibition in the bins next to the 0, because of the little overlap between calls, and a spike of events within 0.5 sec, often over the set confidence interval. Therefore, the calls in the window within ± 0.5 sec. from the focal calls are considered as replies and coloured according to the sex. The replies of the males are depicted in orange and females in grey. Within each cross-correlation the numbers on the top represent the total amount of stack calls over the 8 hours of recording, and the number used to reply to the stack calls of the partner. (PNG 3557 kb)
