# Supplementary material for: Vocal exchanges during pair formation and maintenance in the zebra finch (Taeniopygia guttata)
Source: Front Zool. 2017 Feb 23;14:13. doi: 10.1186/s12983-017-0197-x (PMC5324246; doi:10.1186/s12983-017-0197-x)
Supplement: Additional file 4: — Tables with estimates, Standard Errors (SE), Credible Intervals (CrI) and random factor and residual variance of each LMM used. First is presented model structure, together with mean and SD of the raw data. The graphical representation, if present, is referred after the model. (DOCX 24 kb) [file 12983_2017_197_MOESM4_ESM.docx]

Tables with full model results

Model:

Clumping (556.3±626.8 sec.) ~ Experience * Day + (1|PairID) (Fig. 3A)

| **Fixed effect** | **Estimate** | | **SE** | **95% Credible Intervals** |
| --- | --- | --- | --- | --- |
| Intercept (established and Day1) | 919.8 | | 266.3 | (325.4, 1501.448) |
| Day 3 | 187.6 | | 330.8 | (-543.8, 943) |
| Day 5 | -152.9 | | 330.8 | (-882.4, 581.8) |
| Day 7 | -134.1 | | 330.8 | (-852.8, 601.5) |
| Experience | -890.6 | | 326.2 | (-1600.2, -173.7) |
| Day3: Experience | 474.4 | | 405.1 | (-447.9, 1372) |
| Day5: Experience | 575.4 | | 405.1 | (-329.1, 1458.2) |
| Day7: Experience | 480.9 | | 405.1 | (-421, 1376.2) |
| **Random effects** | | **Variance** | | |
| PairID | | 64905 | | |
| Residual | | 218842 | | |

Model:

|Directionality| (15.7±13.6) ~ Experience * Day + (1|PairID) (Fig. 3B)

| **Fixed effect** | **Estimate** | | **SE** | **95% Credible Intervals** |
| --- | --- | --- | --- | --- |
| Intercept (established and Day1) | 6.5 | | 6.3 | (-6.3, 19.1) |
| Day 3 | 6.2 | | 7 | (-8, 20.3) |
| Day 5 | 5.5 | | 7 | (-9, 19.6) |
| Day 7 | 8.1 | | 7 | (-6.1, 8.2) |
| Experience¹ | 22.7 | | 7.7 | (7.1, 38.3) |
| Day3: Experience² | -16.1 | | 8.6 | (-33.5, 1.6) |
| Day5: Experience² | -21 | | 8.6 | (-38.3, -3.1) |
| Day7: Experience² | -28.1 | | 8.6 | (-45.7, -10.9) |
| **Random effects** | | **Variance** | | |
| PairID | | 58.9 | | |
| Residual | | 98 | | |

Model:

Calling rate (0.1688± 0.1580 calls/sec.) ~ Relative position + (1|Day) + (1|PairID) (Additional file 4)

| **Fixed effect** | **Estimate** | | **SE** | **95% Credible Intervals** |
| --- | --- | --- | --- | --- |
| Intercept | 0.1299¹ | | 0.0535 | (0.0636, 0.2251)¹ |
| Clumping | 0.0476¹ | | 0.0282 | (0.0117, 0.1103)¹ |
| Distance | 0.2042¹ | | 0.0267 | (0.1168, 0.3216)¹ |
| **Random effects** | | **Variance** | | |
| PairID | | 0.0134 | | |
| Day | | 0.0056 | | |
| Residual | | 0.0170 | | |

¹back-transformed estimate of the group (not referenced to the intercept – category “close”)

Model:

Total male calls (0.289±0.261) ~ Total female calls (0.248±0.210) + (1|Day) + (1|Experience/PairID) (Fig.4)

| **Fixed effect** | **Estimate** | | **SE** | **95% Credible Intervals** |
| --- | --- | --- | --- | --- |
| Intercept | 0.190 | | 0.083 | (0.018, 0.362) |
| Total female calls | 0.399 | | 0.164 | (0.066, 0.739) |
| **Random effects** | | **Variance** | | |
| PairID:Experience | | 0.040 | | |
| Day | | 0.007 | | |
| Experience | | 0 | | |
| Residual | | 0.015 | | |

The linear transformation applied is a division by the maximum value, 15501, to transform back to the original scale multiply values in the table by it.

Model:

Male answer calls (0.242±0.230) ~ Female answer calls (0.264±0.273) + (1|Day) + (1|Experience/PairID) (Fig.4)

| **Fixed effect** | **Estimate** | | **SE** | **95% Credible Intervals** |
| --- | --- | --- | --- | --- |
| Intercept | 0.027 | | 0.023 | (-0.020, 0.073) |
| Female answer calls | 0.790 | | 0.039 | (0.711, 0.870) |
| **Random effects** | | **Variance** | | |
| PairID:Experience | | 0.004 | | |
| Day | | 0 | | |
| Experience | | 0 | | |
| Residual | | 0.003 | | |

The linear transformation applied is a division by the maximum value, 2498, to transform back to the original scale multiply values in the table by it.

Model:

Percentage of answers (Males 15.05±10.1 %) ~ Clumping (556.3±626.8 sec.) + (1|Day) + (1| Experience/PairID) (Fig.5)

| **Fixed effect** | **Estimate** | | **SE** | **95% Credible Intervals** |
| --- | --- | --- | --- | --- |
| Intercept | 0 | | 0.2456 | (-0.497, 0.509) |
| Clumping | 0.3001 | | 0.0973 | (0.097, 0.499) |
| **Random effects** | | **Variance** | | |
| PairID:Experience | | 0.5764 | | |
| Day | | 0.0293 | | |
| Experience | | 0 | | |
| Residual | | 0.2368 | | |

Model:

Percentage of answers (Females, 17.7±10.8 %) ~ Clumping (556.3±626.8 sec.) + (1|Day) + (1| Experience/PairID)

| **Fixed effect** | **Estimate** | | **SE** | **95% Credible Intervals** |
| --- | --- | --- | --- | --- |
| Intercept | 0 | | 0.271 | (-0.551, 0.560) |
| Clumping | 0.069 | | 0.119 | (-0.182, 0.315) |
| **Random effects** | | **Variance** | | |
| PairID:Experience | | 0.523 | | |
| Day | | 0.089 | | |
| Experience | | 0 | | |
| Residual | | 0.367 | | |

Model:

Percentage of answers (Males, 16.2± 10.3 %) ~ Clumping (667.6±630.3 sec.) + (1|Day) + (1| Experience /PairID) (model ran only with values of clumping>0, n=40)

| **Fixed effect** | **Estimate** | | **SE** | **95% Credible Intervals** |
| --- | --- | --- | --- | --- |
| Intercept | 0.026 | | 0.258 | (-0.526, 0.574) |
| Clumping | 0.283 | | 0.101 | (0.073, 0.492) |
| **Random effects** | | **Variance** | | |
| PairID:Experience | | 0.647 | | |
| Day | | 0.029 | | |
| Experience | | 0 | | |
| Residual | | 0.196 | | |

**Additional file 4**: Estimates, Standard Errors (SE), Credible Intervals (CrI) and random factor variance of each LMM used. First is presented model structure, together with mean and SD of the raw data. The graphical representation, if present, is referred after the model.
