# Supplementary figures and images for: Vocal exchanges during pair formation and maintenance in the zebra finch (Taeniopygia guttata)
Source: Front Zool. 2017 Feb 23;14:13. doi: 10.1186/s12983-017-0197-x (PMC5324246; doi:10.1186/s12983-017-0197-x)

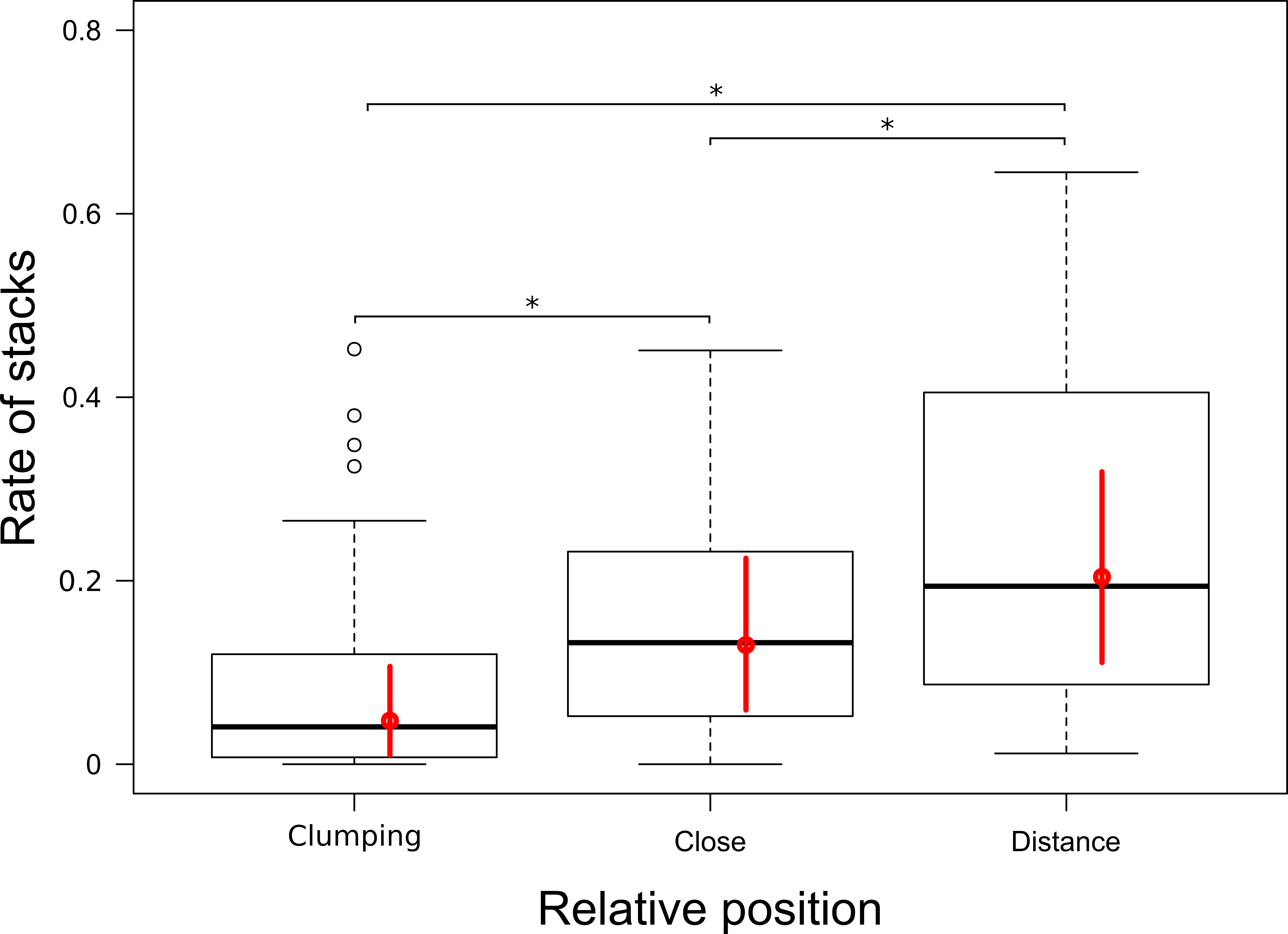

Supplement: Additional file 5: Figure S1. — Rate of stack calls during different relative position of pair members. Rate of stack calls (n/sec) for the 3 different relative positions scored. Clumping: the pair is in physical contact. Close: the space between the birds is less than one bird. Distance: the birds are apart. Boxplots are drawn using raw data, the red dots are the estimated Bayesian values and the red segments the Credible Intervals (CrI) estimated from the LMM.. Both males and females used different rates of stack calls depending on their relative position (data not shown for each sex separately). While in physical contact, (clumping), the birds called the least (measured in calls/sec., mean ± SD, 0.090 ± 0.115 calls/sec., N = 12), followed by close proximity, (close), (0.160 ± 0.126 calls/sec, N = 12); whereas when they were spatially separated, (distance), the pairs had a higher rate of calling (0.243 ± 0.183 calls/sec., N = 12). Using the output of the LMM we calculated the probability that estimated values of one of the relative positions would be higher than the ones of another; asterisks indicate p < 0.05. We found that the probability that the calling rate during “Clumping” was higher than “Close” was p = 0.0074, and “Close” higher than “Distance” was p = 0.0556, indicating strong differences between these categories. Thus, relative position influences the amount of elicited calls. (PNG 333 kb) [file 12983_2017_197_MOESM5_ESM.png]
